# Comparative analysis reveals conservation in genome organization among intestinal *Cryptosporidium* species and sequence divergence in potential secreted pathogenesis determinants among major human-infecting species

**DOI:** 10.1186/s12864-019-5788-9

**Published:** 2019-05-22

**Authors:** Zhixiao Xu, Yaqiong Guo, Dawn M. Roellig, Yaoyu Feng, Lihua Xiao

**Affiliations:** 1 State Key Laboratory of Bioreactor Engineering, School of Resources and Environmental Engineering, East China University of Science and Technology, Shanghai, 200237 China; 2 Key Laboratory of Zoonosis of Ministry of Agriculture, College of Veterinary Medicine, South China Agricultural University, Guangzhou, 510642 China; 3 Division of Foodborne, Waterborne, and Environmental Diseases, National Center for Emerging and Zoonotic Infectious Diseases, Centers for Disease Control and Prevention, Atlanta, GA 30329 USA

**Keywords:** *Cryptosporidium* chipmunk genotype I, Comparative genomics, MEDLE family proteins, Insulinase-like proteases, ABC transporters

## Abstract

**Background:**

Cryptosporidiosis is a major cause of gastrointestinal diseases in humans and other vertebrates. Previous analyses of invasion-related proteins revealed that *Cryptosporidium parvum*, *Cryptosporidium hominis*, and *Cryptosporidium ubiquitum* mainly differed in copy numbers of secreted MEDLE proteins and insulinase-like proteases and sequences of mucin-type glycoproteins. Recently, *Cryptosporidium* chipmunk genotype I was identified as a novel zoonotic pathogen in humans. In this study, we sequenced its genome and conducted a comparative genomic analysis.

**Results:**

The genome of *Cryptosporidium* chipmunk genotype I has gene content and organization similar to *C. parvum* and other intestinal *Cryptosporidium* species sequenced to date. A total of 3783 putative protein-encoding genes were identified in the genome, 3525 of which are shared by *Cryptosporidium* chipmunk genotype I and three major human-pathogenic *Cryptosporidium* species, *C. parvum*, *C. hominis*, and *Cryptosporidium meleagridis*. The metabolic pathways are almost identical among these four *Cryptosporidium* species. Compared with *C. parvum*, a major reduction in gene content in *Cryptosporidium* chipmunk genotype I is in the number of telomeric genes encoding MEDLE proteins (two instead of six) and insulinase-like proteases (one instead of two). Highly polymorphic genes between the two species are mostly subtelomeric ones encoding secretory proteins, most of which have higher dN/dS ratios and half are members of multiple gene families. In particular, two subtelomeric ABC transporters are under strong positive selection.

**Conclusions:**

*Cryptosporidium* chipmunk genotype I possesses genome organization, gene content, metabolic pathways and invasion-related proteins similar to the common human-pathogenic *Cryptosporidium* species, reaffirming its human-pathogenic nature. The loss of some subtelomeric genes encoding insulinase-like proteases and secreted MEDLE proteins and high sequence divergence in secreted pathogenesis determinants could contribute to the biological differences among human-pathogenic *Cryptosporidium* species.

**Electronic supplementary material:**

The online version of this article (10.1186/s12864-019-5788-9) contains supplementary material, which is available to authorized users.

## Background

*Cryptosporidium* spp. are important apicomplexan parasites, causing moderate to severe diarrhea in humans and various animals. Currently, there are near 40 named *Cryptosporidium* species and about the same number of genotypes with unknown species status [1]. Among them, approximately 20 have been found in humans [2]. However, *Cryptosporidium parvum* and *Cryptosporidium hominis* are two major species infecting humans. Other species, including *Cryptosporidium meleagridis*, *Cryptosporidium felis*, *Cryptosporidium canis*, *Cryptosporidium ubiquitum*, *Cryptosporidium cuniculus*, *Cryptosporidium viatorum*, and *Cryptosporidium muris*, are less common [1].

*Cryptosporidium* species differ in host range and public health significance [3]. Among the human-pathogenic species, *C. parvum* has the broadest host range. In addition to humans, it infects ruminants, equine animals, rodents, and some other animals. In contrast, *C. hominis* is mostly restricted to humans, nonhuman primates, and equine animals [1]. As the third most prevalent species infecting humans, *C. meleagridis* has been reported in both mammals and birds [2, 4, 5]. Another *Cryptosporidium* species, *C. ubiquitum,* also has a broad host range, being commonly detected in small ruminants, rodents, in addition to humans [6, 7]. *Cryptosporidium* chipmunk genotype I, which was initially found in several species of rodents, is a novel zoonotic pathogen, having been reported in humans recently [8, 9]. It is one of the three major zoonotic *Cryptosporidium* species in humans in rural United States [10].

Results of comparative genomics analysis suggest that members of several secreted protein families, such as MEDLE proteins, insulinase-like proteases, and mucin-type glycoproteins, are potential determinants for differences in host range among *Cryptosporidium* species [11, 12]. The difference in the number of MEDLE genes among *Cryptosporidium* species or *C. parvum* subtype families (IIa in bovines and IId in small ruminants) indicates that MEDLE proteins could contribute to differences in host specificity [11, 13]. Insulinase-like proteases are secreted proteases, being involved in processing invasion-related proteins in apicomplexans or modifying host cell proteins [14]. Mucin-type glycoproteins are known to be involved in the attachment and invasion of *Cryptosporidium* spp. [15]. Compared with *C. parvum*, a reduction in the numbers of genes encoding the MEDLE family secreted proteins and insulinase-like proteases was seen in the 3′ subtelomeric regions of chromosomes 5 and 6 of the *C. hominis* genome [11]. The orthologous regions encoding subtelomeric insulinases and MEDLE proteins are entirely absent in the genomes of *C. ubiquitum* and gastric species *Cryptosporidium andersoni* [12]. In addition to the gene losses, genetically related *Cryptosporidium* species differ significantly in sequences of mucin-type glycoproteins [11, 12]. As intestinal and gastric *Cryptosporidium* species differ significantly in the numbers and sequences of genes encoding mucin-type glycoproteins and insulinase-like proteases, these proteins and other secreted pathogenesis determinants (SPDs) potentially play an important role in tissue tropism also [12].

Although the genomes of several *Cryptosporidium* species have been sequenced recently, we still have very limited knowledge of genome evolution among *Cryptosporidium* spp. [16, 17]. In this study, we have sequenced the genome of *Cryptosporidium* chipmunk genotype I and conducted a comparative genomic analysis of eight *Cryptosporidium* species that have been sequenced thus far [11, 12, 18–20].

## Results

### Genome features

**Fig. 1:**
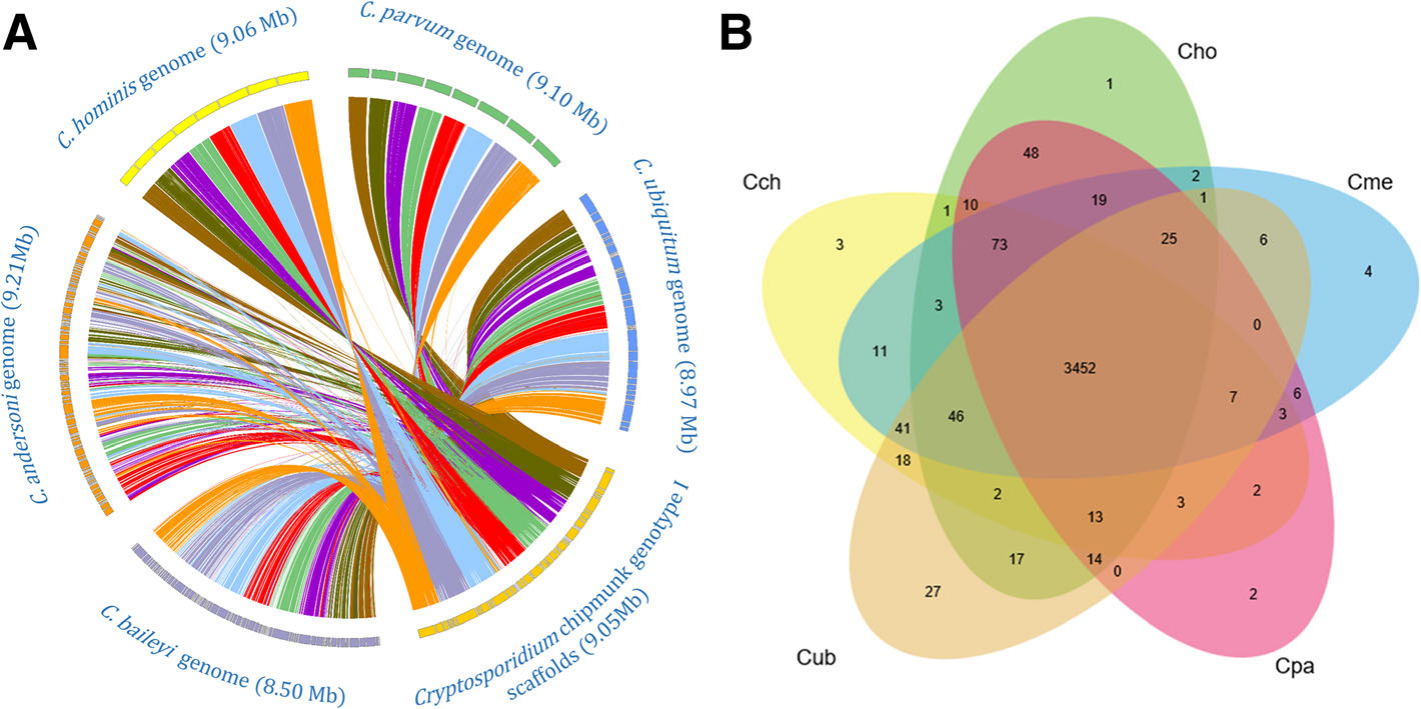
Syntenic relationship and shared orthologous genes among *Cryptosporidium* spp. **a** Syntenic relationship in gene organization among genomes of *Cryptosporidium* chipmunk genotype I, *Cryptosporidium parvum*, *C. hominis*, *C. ubiquitum*, *C. baileyi*, and *C. andersoni*. Syntenic blocks (regions with orthologous genes) are connected with lines, with the colors representing 8 chromosomes of *C. parvum*. **b** Venn diagram of orthologous genes shared by five *Cryptosporidium* spp. Abbreviations of taxa: *Cryptosporidium parvum* IOWA (Cpa); *C. hominis* Ude (Cho); *C. meleagridis* (Cme); *Cryptosporidium* chipmunk genotype I (Cch); *C. ubiquitum* (Cub)

We generated 6.8 million 250-bp paired-end reads from one *Cryptosporidium* chipmunk genotype I isolate 37,763 from a naturally infected person in the United States by Illumina sequencing. After filtering out contigs from contaminants among the 298 initial contigs generated using the CLC Genomics Workbench, we assembled a *Cryptosporidium* genome of 9.05 Mb in 50 contigs (without any scaffolding during the processing), with an estimated 188-fold coverage and an N50 of 320,570 bp. We combined gene prediction results obtained from Augustus, Geneid, and Genemark, leading to the identification of 3783 protein-encoding genes. At the genome level, *Cryptosporidium* chipmunk genotype I has high nucleotide and amino acid sequence identity to *C. parvum* (82.25 and 83.49%, respectively), *C. hominis* (82.48 and 83.99%, respectively), and *C. meleagridis* (81.22 and 81.68%, respectively; Table 1). Among the eight *Cryptosporidium* species with whole genome sequence data, *Cryptosporidium* chipmunk genotype I has the highest GC content in the overall genome (32.0%) and coding regions (33.6%). The genome of *Cryptosporidium* chipmunk genotype I has near complete sequence synteny with that of *C. parvum* and *C. ubiquitum* (Fig. 1a), with a rearrangement of ~ 126 kb between *Cryptosporidium* chipmunk genotype I and *C. parvum*. The 5′ subtelomeric region of chromosome 6 in *Cryptosporidium* chipmunk genotype I, which contains 52 genes, is translocated with the 5′ subtelomeric region of chromosome 8 containing 53 genes (*cgd8_10*~*cgd8_530*) in *C. parvum*. This rearrangement was observed in both assemblies produced by the CLC Genomics Workbench and the SPAdes assembler. Advanced sequencing using the PacBio technology is needed to confirm the existence of this genome rearrangement. Lower synteny was seen with genomes of *C. baileyi* and *C. andersoni. Cryptosporidium* chipmunk genotype I shares almost the same gene density and number of tRNA genes with other *Cryptosporidium* spp. It, however, has gene content slightly lower than *C. parvum* and *C. hominis*, but similar to *C. meleagridis*, *C. ubiquitum*, and *C. baileyi* (Table 1).

**Table 1:** Genomic features of *Cryptosporidium* chipmunk genotype I in comparison with some other *Cryptosporidium* spp

	*Cryptosporidium* chipmunk genotype I	*C. parvum*	*C. hominis* UdeA01	*C. meleagridis*	*C. ubiquitum*	*C. baileyi*	*C. andersoni*	*C. muris*
Total length (Mb)	9.05	9.1	9.06	8.97	8.97	8.5	9.09	9.21
No. of super contigs	50	8	97	57	27	153	135	45
GC content (%)	32	30.3	30.1	31	30.8	24.3	28.5	28.4
Nucleotide sequence identity (%)	–	82.25	82.48	81.22	78.65	46.08	26.44	26.89
Number of genes	3783	3805	3819	3782	3767	3728	3905	3937
Total length of CDS (Mb)	6.94	6.83	6.81	6.91	6.94	6.69	6.86	6.93
GC content in CDS (%)	33.6	31.9	31.8	32.4	33	25.6	30.1	30
Amino acid sequence identity (%)	–	83.49	83.99	81.68	79.04	58.89	47.03	47.22
GC content at 3^rd^ position in codons (%)	26.9	22.5	23.5	24.1	24.5	12.6	18.1	17.8
Gene density (gene/Mb)	418	418.1	421.5	421.6	420	438.6	429.6	427.5
Percent coding (%)	76.7	75	75.2	77	77.4	78.7	75.5	75.2
No. of genes with intron	515	163	417	506	758	763	832	798
Genes with intron (%)	13.6	4.2	10.9	13.4	20.1	20.5	21.3	20.3
No. of tRNA	45	45	45	45	45	46	44	45
No. of tRNA^met^	2	2	2	2	2	2	2	2
Proteins with signal peptide	396	397	391	397	399	344	309	323
Proteins with transmembrane domain	793	832	817	805	772	813	839	836
Proteins with GPI anchor	57	63	54	55	50	57	47	52

**Fig. 2:**
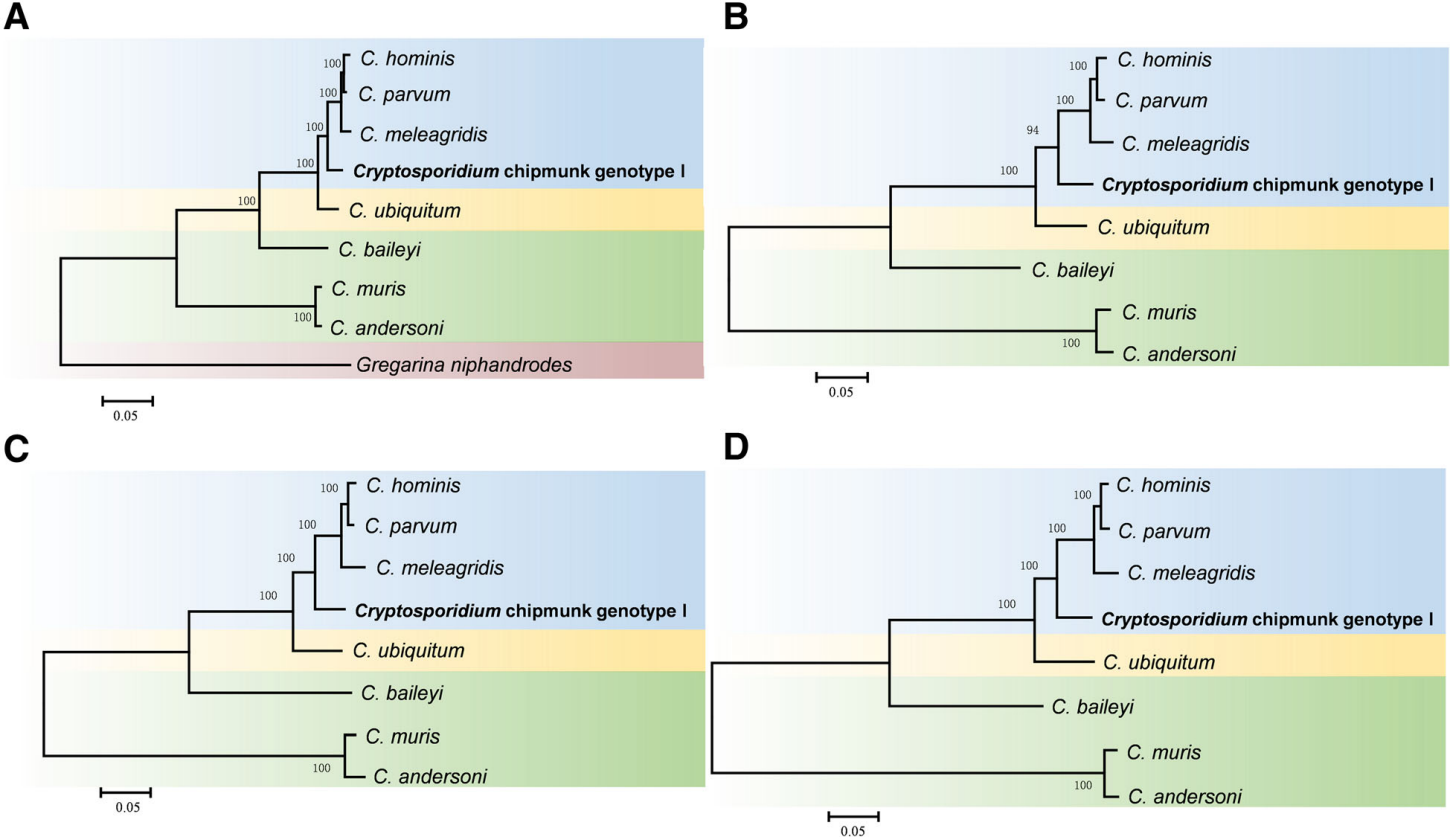
Phylogenetic relationship of *Cryptosporidium* spp. **a** Phylogenetic relationship of *Cryptosporidium* spp. based on maximum likelihood analysis of sequences of 100 shared proteins. **b** Phylogenetic relationship of *Cryptosporidium* spp. based on maximum likelihood analysis of TRAP sequences. **c** Phylogenetic relationship of *Cryptosporidium* spp. based on maximum likelihood analysis of mucin-type glycoproteins. **d** Phylogenetic relationship of *Cryptosporidium* spp. based on maximum likelihood analysis of insulinase-like proteases

Orthology delineation identified only a small number of species-specific genes among eight *Cryptosporidium* spp. Approximately 3525 genes are shared by *C. parvum*, *C. hominis*, *C. meleagridis*, and *Cryptosporidium* chipmunk genotype I (Fig. 1b). There are only three *Cryptosporidium* chipmunk genotype I-specific genes. One of them was identified as an insulinase-like protease, but the functions of other two genes are unknown. Phylogenetic analysis of amino acid sequences from 100 orthologous genes supported the close relatedness of *Cryptosporidium* chipmunk genotype I to these human-pathogenic *Cryptosporidium* species (Fig. 2a).

**Fig. 3:**
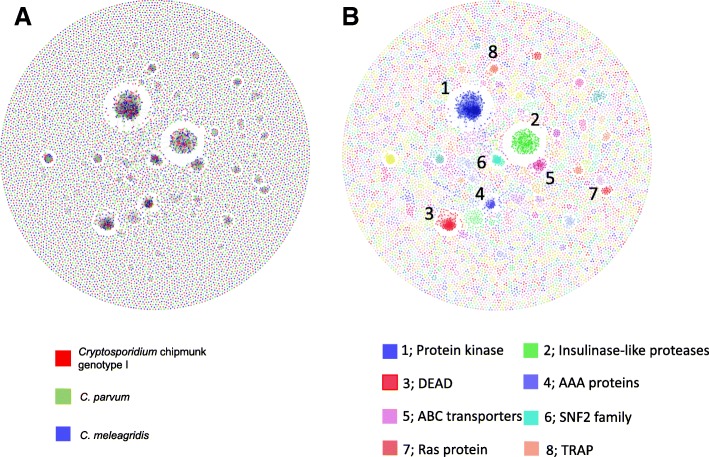
Protein architecture network based on sequence similarity of all proteins in proteomes of *Cryptosporidium* chipmunk genotype I, *Cryptosporidium parvum*, and *Cryptosporidium meleagridis*. **a** Proteins of *Cryptosporidium* chipmunk genotype I, *C. parvum*, and *C. meleagridis*, represented by the colors red, green, and blue, respectively. **b** Identity of major clusters in the *Cryptosporidium* proteome

Multiple gene families are present in *Cryptosporidium* chipmunk genotype I as well as other *Cryptosporidium* species. Protein architecture network analysis of *Cryptosporidium* chipmunk genotype I, *C. parvum*, and *C. meleagridis* revealed the existence of several clusters (Fig. 3a). Two of the major clusters (1 and 2) in the network consisted of protein kinases and insulinase-like peptidases of the three *Cryptosporidium* species. There are 75, 79, and 78 genes encoding protein kinases in *Cryptosporidium* chipmunk genotype I, *C. parvum*, and *C. meleagridis*, respectively. *C. parvum* possesses 23 genes encoding insulinase-like peptidases, while 22 genes encoding insulinase-like peptidases was detected *Cryptosporidium* chipmunk genotype I and *C. meleagridis*. Members of helicases such as DEAD and SNF2 formed Clusters 3 and 6, which are involved in unwinding nucleic acids and RNA metabolism. The three *Cryptosporidium* species possess the same number of genes encoding DEAD (39 genes) and SNF2 (16 genes). ATPases associated with diverse cellular activities (AAA) and ATP-binding cassette (ABC) transporters formed Cluster 4 and 5. We found 21 genes encoding ABC transporters in all three species. Compared with *C. parvum* and *C. meleagridis*, one gene encoding AAA proteins was lost in *Cryptosporidium* chipmunk genotype I (24 AAA proteins). In addition, the Ras proteins, which are involved in- intracellular signaling, formed Cluster 7. Furthermore, the 12 thrombospondin-related adhesive proteins (TRAPs), which are presumably microneme proteins present in all three *Cryptosporidium* species under analysis [21, 22], are included in Cluster 8 (Fig. 3b).

### Characteristics of metabolism in *Cryptosporidium* chipmunk genotype I

#### Carbohydrate metabolism

Similar to other intestinal *Cryptosporidium* spp., *Cryptosporidium* chipmunk genotype I lacks genes encoding core enzymes of the tricarboxylic acid (TCA) cycle, but possesses enzymes for the synthesis of pyruvate from glucose in glycolysis. Furthermore, a gene for a phosphoenolpyruvate carboxylase (Cch_34.2917) was detected in *Cryptosporidium* chipmunk genotype I, suggesting that this parasite can convert phosphoenolpyruvate (PEP) to oxaloacetate (OAA).

Like other *Cryptosporidium* spp., *Cryptosporidium* chipmunk genotype I lacks genes encoding enzymes for de novo isoprenoid biosynthesis. Two genes encoding farnesyl diphosphate (FPP) synthase (Cch_19.1677) and polyprenyl synthase (Cch _17.1265) were detected in *Cryptosporidium* chipmunk genotype I. These two genes were shown transcribed in *C. parvum* in vitro [23], but are absent in *C. ubiquitum* [12].

#### Electron transport chain

**Fig. 4:**
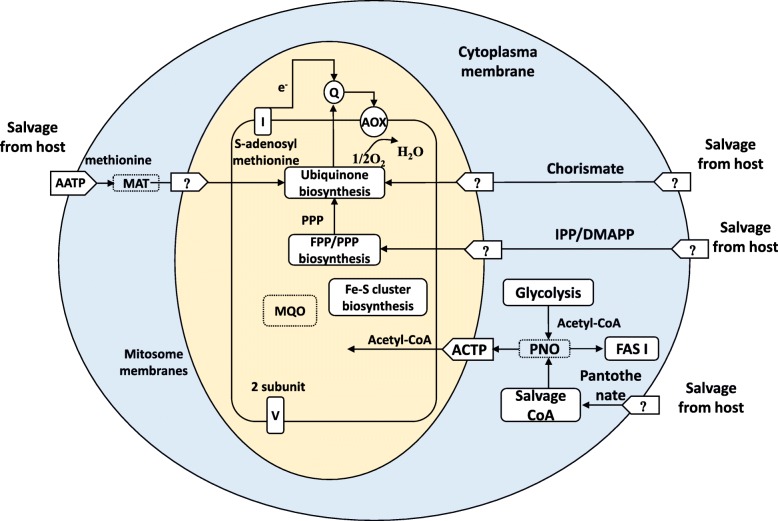
Mitochondrial metabolism of *Cryptosporidium* chipmunk genotype I. Abbreviation of enzymes: AOX: alternative oxidase; PNO: pyruvate:NADP(+) oxidoreductase; MAT: methionine adenosyl transferase; MQO: malate:quinone oxidoreductase. Abbreviation of metabolites: Q: ubiquinone (coenzyme Q); CoA: coenzyme A; IPP: isopentenyl diphosphate; DMAPP: dimethylallyl diphosphate; FPP: farnesyl diphosphate; PPP: polyprenyl diphosphate. Abbreviation of transporter proteins: AATP: amino acids transporter protein; ACTP: acetyl-CoA transporter protein

A progressive reduction in the electron transport chain was reported in *Cryptosporidium* spp. [12]. Most intestinal *Cryptosporidium* spp. have an alternative oxidase (AOX) and a reduced conventional electron transport system, except for *C. ubiquitum*, which does not have them and the AOX. Unlike *C. ubiquitum*, *Cryptosporidium* chipmunk genotype I and the three major human-pathogenic species possess all enzymes and proteins involved in the ubiquinone biosynthesis (Fig. 4).

The number of mitochondrial carrier proteins in *Cryptosporidium* spp. is in agreement with the nature of the electron transport system. As reported previously [12], gastric *Cryptosporidium* spp. have more mitochondrial carrier proteins than intestinal *Cryptosporidium* spp. (Table 3). Among the latter, eight mitochondrial carrier proteins were detected in *Cryptosporidium* chipmunk genotype I and *C. meleagridis*, compared with nine in *C. parvum* and *C. hominis* and six in *C. ubiquitum* and *C. baileyi*, which also does not have the AOX (Table 3). These data indicate that the mitosome metabolic capability in *Cryptosporidium* chipmunk genotype I is similar to that in the three major human-pathogenic *Cryptosporidium* species.

#### Nucleotide metabolism

All *Cryptosporidium* spp. cannot synthesize purine rings or pyrimidines de novo (Table 2). Instead, they must salvage these nucleotides from the host via the nucleoside transporter (Table 3). However, the enzymes involved in the inter-conversion of purines and pyrimidines are different among *Cryptosporidium* species. The gene encoding the guanosine monophosphate (GMP) synthase (*cgd5_4520* in *C. parvum*) is lost in *Cryptosporidium* chipmunk genotype I, indicating that *Cryptosporidium* chipmunk genotype I cannot convert xanthosine 5′-phosphate (XMP) to GMP. Furthermore, the last gene (*cgd1_3860*) in chromosome 1 of *C. parvum*, which encodes a deoxyuridine triphosphate (dUTP) diphosphatase, has an ortholog in *C. hominis* (*Chro.10434*), but is absent in *Cryptosporidium* chipmunk genotype I and *C. meleagridis* (Additional file 1: Table S1). The ortholog of another dUTP diphosphatase gene in *C. parvum* (*cgd7_5170*), however, is present in *Cryptosporidium* chipmunk genotype I (*Cch_42.3131*).

**Table 2:** Comparison of essential metabolic pathways among *Cryptosporidium* spp. and some other common apicomplexan parasites

Category	Metabolic pathway	Cchi	Cpar	Chom	Cmel	Cubi	Cbai	Cand	Pfal	Tgon
Carbohydrate and energy metabolism	Glycolysis	+	+	+	+	+	+	+	+	+
	Methylcitrate cycle	–	–	–	–	–	–	–	–	+
	TCA cycle	–	–	–	–	–	–	+	+	+
	Pentose phosphate pathway	–	–	–	–	–	–	–	+	+
	Shikimate biosynthesis	–	–	–	–	–	–	–	+	+
	Folate biosynthesis	–	–	–	–	–	–	–	+	+
	Synthesis of pterin	–	–	–	–	–	–	–	–	+
	Galactose metabolism	–	–	–	–	–	–	–	–	+
	Synthesis of starch	+	+	+	+	+	+	+	–	+
	Synthesis of trehalose	+	+	+	+	+	+	+	–	+
	Synthesis of 1,3-beta-glucan	–	–	–	–	–	–	–	–	+
	Conversion between UDP-Glc and UDP-Gal	+	+	+	+	+	+	+	–	+
	Conversion between GDP-Man and GDP-Fuc	–	–	–	–	–	–	–	+	+
	Conversion from UDP-Glc to UDP-GlcA to UDP-Xyl	+	+	+	+	+	+	+	–	–
	Synthesis of mannitol from fructose	+	+	+	+	+	+	+	–	–
	Fatty acid biosynthesis in cytosol (FAS I)	+	+	+	+	+	+	+	–	+
	Fatty acid biosynthesis in apicoplast (FAS II)	–	–	–	–	–	–	–	+	+
	Fatty acid degradation	–	–	–	–	–	–	–	–	+
	Oxidative phosphorylation (NADH dehydrogenase)	+	+	+	+	+	+	+	+	+
	Oxidative phosphorylation (Complex II)	–	–	–	–	–	–	+	+	+
	Oxidative phosphorylation (Complex III)	–	–	–	–	–	–	1 sub	+	+
	Oxidative phosphorylation (Complex IV)	–	–	–	–	–	–	–	+	+
	F-ATPase	2 sub	2 sub	2 sub	2 sub	2 sub	2 sub	+	+	+
	Alternative oxidase (AOX)	+	+	+	+	–	–	+	–	–
	Glyoxalase metabolism producing D-lactate	–	–	–	–	–	–	–	+	+
	Synthesis of isoprene (MEP/DOXP)	–	–	–	–	–	–	–	+	+
	Synthesis of farnesyl/polyprenyl diphosphate	+	+	+	+	–	–	+	+	+
Nucleotide metabolism	Synthesis of purine rings de novo	–	–	–	–	–	–	–	–	–
	Conversion from IMP to XMP	+	+	+	+	+	–	–	+	+
	Conversion from XMP to GMP	–	+	+	–	–	–	–	+	+
	Synthesis of pyrimidine de novo	–	–	–	–	–	–	–	+	+
Amino acid metabolism	Synthesis of alanine from pyruvate	–	–	–	–	–	–	–	–	+
	Synthesis of glutamate from nitrite/nitrate	–	–	–	–	–	–	–	+	+
	Conversion from glutamate to glutamine	+	+	+	+	+	+	+	+	+
	Synthesis of aspartate from oxaloacetate and glutamate	–	–	–	–	–	–	–	+	+
	Conversion from aspartate to asparagine	+	+	+	+	+	–	–	+	+
	Conversion from glutamate to proline	+	+	+	+	+	+	+	–	+
	Synthesis of serine from glycerate/glycerol phosphate	–	–	–	–	–	–	–	–	+
	Conversion from serine to cysteine	–	–	–	–	–	–	–	–	+
	Conversion from serine to glycine	+	+	+	+	+	+	+	+	+
	Recycle homocysteine into methionine	–	–	–	–	–	–	–	+	+
	Synthesis of lysine from aspartate	–	–	–	–	–	–	–	–	+
	Synthesis of threonine from aspartate	–	–	–	–	–	–	–	–	+
	Synthesis of ornithine from arginine	–	–	–	–	–	–	–	+	–
	Synthesis of ornithine from proline	–	–	–	–	–	–	–	+	+
	Synthesis of polyamine from ornithine	–	–	–	–	–	–	–	+	–
	Polyamine pathway backward	+	+	+	+	+	+	+	–	+
	Degradation of branch-chain amino acids	–	–	–	–	–	–	–	–	+
	Synthesis of tryptophan	+	+	+	–	+	–	–	–	–
	Aromatic amino acid hydroxylases (AAAH)	–	–	–	–	–	–	–	–	+
Vitamin and others	Synthesis of ubiquinone (Coenzyme Q)	+	+	+	+	–	–	+	+	+
	Synthesis of Fe-S cluster	+	+	+	+	+	+	+	+	+
	Synthesis of heme	–	–	–	–	–	–	–	+	+
	Synthesis of thiamine (Vitamin B1)	–	–	–	–	–	–	–	+	–
	Conversion from thiamine to thiamine pyrophosphate (TPP)	–	–	–	–	–	–	–	+	+
	Synthesis of FMN/FAD from riboflavin	–	–	–	–	–	–	–	+	+
	Synthesis of pyridoxal phosphate (Vitamin B6) de novo	–	–	–	–	–	–	–	+	+
	Synthesis of NAD(P) + de novo from nicotinate/nicotinamide	–	–	–	–	–	–	–	+	+
	Synthesis of pantothenate from valine	–	–	–	–	–	–	–	–	+
	Synthesis of CoA from pantothenate	+	+	+	+	+	+	+	+	+
	Synthesis of lipoic acid de novo in apicoplast	–	–	–	–	–	–	–	+	+
	Salvage of lipoic acid in mitochondria	–	–	–	–	–	–	+	+	+
	Synthesis of porphyrin/cytochrome proteins	–	–	–	–	–	–	–	+	+

Plus symbols denote that these metabolic pathways were identified in this apicomplexan parasite, whereas minus symbols denote that these metabolic pathways are absent from this apicomplexan parasite. *Abbreviation*: *Cchi Cryptosporidium* chipmunk genotype I, *Cpar Cryptosporidium parvum*, *Chom C. hominis, Cmel C. meleagridis*, *Cubi C. ubiquitum*, *Cbai C. baileyi*, *Cand C. andersoni*, *Pfal Plasmodium falciparum, Tgon Toxoplasma gondii*

**Table 3:** Putative transporters in *Cryptosporidium* spp. and some other common apicomplexan parasites^a^

Substrates	Cellular location	Cchi	Cpar	ChomUde	Cmel	Cubi	Cbai	Cand	Cmur	Pfal	Tgon
Hexose		2	2	2	2	2	2	2	3	2	5
Triose phosphate	Plasma/Apicoplast membrane	7	8	8	8	8	7	8	8	4	4
Amino acids	Plasma membrane	10	10	10	10	10	10	12	12	1	6
Nucleobase/nucleoside	Plasma membrane	1	1	1	1	1	1	1	1	4	4
Nucleotide-sugar	Plasma membrane	3	3	3	3	3	2	2	2	1	4
Folate/pterine	Plasma membrane	1	1	2	1	1	1	1	1	2	7
Formate/nitrite		0	0	0	0	0	0	0	0	1	3
GABA (aminobutanoate)	Plasma/Mitochondrial membrane	0	0	0	0	0	0	0	0	2	5
Acetyl-CoA		1	1	1	1	1	1	1	1	1	1
Chloride		0	0	0	0	0	0	0	0	0	2
Inorganic phosphate		0	0	0	0	0	0	0	0	1	1
Sulfate		1	1	1	1	1	1	1	1	1	4
Sodium/potassium/calcium		2	2	2	2	2	2	3	3	0	9
Zinc		2	2	2	2	2	2	2	2	2	4
Copper		1	1	1	1	1	1	1	1	2	3
Choline	Plasma membrane	0	0	0	0	0	0	0	0	1	2
Cadmium/zinc/cobalt (efflux)	Plasma membrane	1	1	1	1	1	1	1	1	1	1
Glycerol/water	Plasma membrane	0	0	0	0	0	0	0	0	2	2
ABC transporter	Plasma membrane	21	21	21	21	21	22	21	21	16	24
Mitochondrial carrier	Mitochondrial membrane	8	9	9	8	6	6	13	12	14	21

*Cchi Cryptosporidium* chipmunk genotype I, *Cpar Cryptosporidium parvum*, *ChomUde C. hominis* UdeA01, *Cmel C. meleagridis*, *Cubi C. ubiquitum*, *Cbai C. baileyi*, *Cand C. andersoni*, *Cmur C. muris*, *Pfal Plasmodium falciparum*, *Tgon Toxoplasma gondii*

^a^The detection of these transporter proteins was based on the Pfam search results

### N-glycan and GPI-anchor precursors in *Cryptosporidium* chipmunk genotype I

A secondary loss of *Alg* genes in asparagine (N)-linked glycosylation was reported in apicomplexans [24]. The biosynthesis of N-glycans is different not only among apicomplexan parasites but also within the genus *Cryptosporidium*. Similar to *C. hominis*, *C. parvum*, *C. meleagridis*, and *C. ubiquitum*, *Cryptosporidium* chipmunk genotype I possesses nine sugars in N-glycan precursors, compared to eight sugars in *C. baileyi* and five in *C. andersoni*.

In glycosylphosphatidylinositol (GPI) anchor biosynthesis, the essential phosphatidylinositol glycan (PIG)-B was detected in *Cryptosporidium* chipmunk genotype I but lost in *C. ubiquitum*. Similar to other *Cryptosporidium* spp., genes encoding PIG-W and glycosylphosphatidylinositol deacylase (PGAP1) involved in the acylation and de-acylation of inositol are absent in *Cryptosporidium* chipmunk genotype I.

### Characteristics of invasion-related proteins in *Cryptosporidium* chipmunk genotype I

**Fig. 5:**
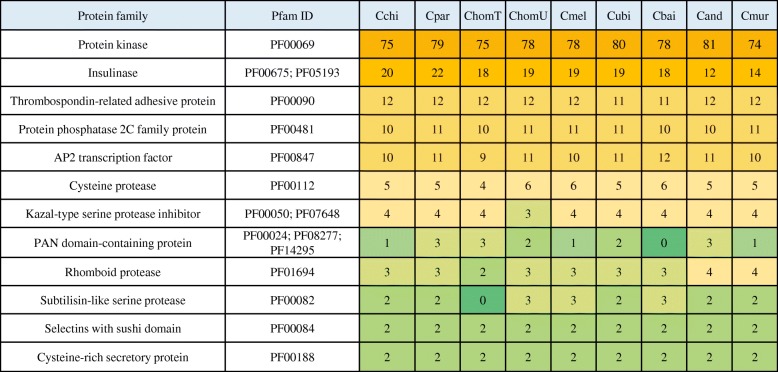
Comparison of major invasion-related protein families among *Cryptosporidium* species. The number of each protein family was identified based on Pfam domain search. The dark yellow cell represents the largest number of members in the protein families while the green cell represents the smallest number of members in the protein families in *Cryptosporidium* spp. Because of highly-fragmented draft genome of *C. hominis* TU502, some Pfam domains were not detected in *C. hominis* but observed in *C. hominis* UKH1. Abbreviations of taxa: *Cryptosporidium* chipmunk genotype I (Cchi); *Cryptosporidium parvum* (Cpar); *C. hominis* TU502 (ChomT); *C. hominis* UKH1 (ChomU); *C. meleagridis* (Cmel); *C. ubiquitum* (Cubi); *C. baileyi* (Cbai); *C. andersoni* (Cand); *C. muris* (Cmur)

*Cryptosporidium* chipmunk genotype I and other intestinal *Cryptosporidium* spp. possess similar numbers and components of major protein families, including some of those involved in invasion, such as protein kinases and TRAPs. *Cryptosporidium* species, however, differ in the number of genes encoding other invasion-related proteins, such as insulinase-like peptidases, MEDLE secretory proteins, and mucin glycoproteins. For example, gastric species *C. andersoni* and *C. muris* have fewer genes encoding insulinase-like peptidases (Fig. 5). Compared with *C. parvum*, two of the 23 insulinase-like protease genes and four of the six MEDLE family protein genes are lost in *Cryptosporidium* chipmunk genotype I, all located at the subtelomeric regions of chromosomes 5 and 6 (Additional file 1: Table S2). A new gene (*Cch_105.391*) of the insulinase gene family, which has significant sequence similarity to *cgd3_4260*, was detected at the 5′ end of chromosome 7 (contig_105). Furthermore, all three major human-infecting species, *C. parvum*, *C. hominis*, and *C. meleagridis*, possess MEDLE protein genes, but none of them were observed in *C. ubiquitum*, *C. baileyi, C. andersoni*, or *C. muris* (Additional file 1: Table S2).

Comparisons of mucin-type glycoproteins among eight *Cryptosporidium* species had shown a high divergence between human-infecting and animal-infecting species. The gp60/40/15 complex, which is a single-copy gene in *Cryptosporidium* chipmunk genotype I, is absent in *C. andersoni* and *C. muris*, but has 7 paralogous genes in two clusters in *C. baileyi*. *Cryptosporidium* chipmunk genotype I possesses a series of mucin-type glycoproteins, such as CP2, but many of them are absent in *C. baileyi, C. andersoni*, or *C. muris* (Additional file 1: Table S2). Phylogenetic analysis of invasion-related proteins, including mucin-type glycoproteins, insulinase-like proteases and TRAPs, confirmed the close relatedness of *Cryptosporidium* chipmunk genotype I to human-infecting species (Fig. 2b-d).

### Other genes gains and losses in *Cryptosporidium* chipmunk genotype I

Compared with other related *Cryptosporidium* spp., gains and losses of several other genes were detected in *Cryptosporidium* chipmunk genotype I. One 4500-bp insertion, which contains a *Cryptosporidium* chipmunk genotype I-specific gene (*Cch_13.573*) was seen at the 3′ end of chromosome 4. In the large insertion at the 3′ end of chromosome 5 (contig_35) in *Cryptosporidium* chipmunk genotype I, *Cch_35.2955* is a paralog of *Cch_40.3117*, *Cch_7.3568* and *Cch_1.1*. Six members (*Chro.00007*, *Chro.60010*, *Chro.60630*, *Chro.80010*, *Chro.60631*, and *Chro.60634*) of this gene family were detected in *C. hominis* but only three (*cgd5/6_5500*, *cgd6_5500*, and *cgd8_10*) were detected in *C. parvum*. In contrast, the ortholog of *cgd4_3690*, which encodes a low complexity protein with a large glycine-rich repeat, was lost in *Cryptosporidium* chipmunk genotype I. The same is also true for the gene for a cysteine-rich protein with a signal peptide in *C. parvum* (*cgd4_4500*), *C. hominis* (*Chro.40511*), and *C. meleagridis* (*C_mele_24106.404*). Similar to *C. hominis* and *C. meleagridis*, *Cryptosporidium* chipmunk genotype I has only one copy of the paralogous genes *cgd8_660_670* and *cgd8_680_690*. Similarly, orthologs of *cgd4_10*, *cgd7_5530*, *cgd8_4180* and *cgd8_5420* were not detected in *Cryptosporidium* chipmunk genotype I (Additional file 1: Table S1). They are mostly subtelomeric genes encoding hypothetical proteins. Among 23 genes lost in *Cryptosporidium* chipmunk genotype I, 11 encode proteins with signal peptides (*cgd4_10*, *cgd4_4500*, *cgd7/5_4510*, *cgd7/5_4530*, *cgd7/5_4590*, *cgd5/6_5480*, *cgd5/6_5490*, *cgd5/6_5520–5510*, *cgd6_5520–5510*, *cgd7_1280*, *cgd8_660_70*) and 19 are located in the subtelomeric regions (*cgd1_3860*, *cgd3_370*, *cgd4_10*, *cgd4_3690*, *cgd4_4500*, *cgd5/6_5490*, *cgd5/6_5520–5510*, *cgd6_5500*, *cgd6_5520–5510*, *cgd7/5_4580*, *cgd7/5_4590*, *cgd7/5_4610*, *cgd7/5_4510*, *cgd7/5_4520*, *cgd7/5_4530*, *cgd7_5530*, *cgd8_10*, *cgd8_660_70*, *cgd8_5420*).

### Highly divergent genes between *Cryptosporidium* chipmunk genotype I and *Cryptosporidium parvum*

The putative proteome of *Cryptosporidium* chipmunk genotype I was compared with the annotated protein-encoding genes of *C. parvum* and *C. ubiquitum*. We found 49 highly divergent genes between *Cryptosporidium* chipmunk genotype I and these two *Cryptosporidium* species with an amino acid identity below 65% (Additional file 1: Table S3). Among them, 43 (87.8%) genes encode proteins with signal peptides, 41 (84.9%) are located in the subtelomeric regions, and 25 (51.0%) possess paralogous genes. Many of the genes encode mucins, *Cryptosporidium*-specific SKSR or FLGN families, and low complexity proteins.

### Genes under selection pressure

**Fig. 6:**
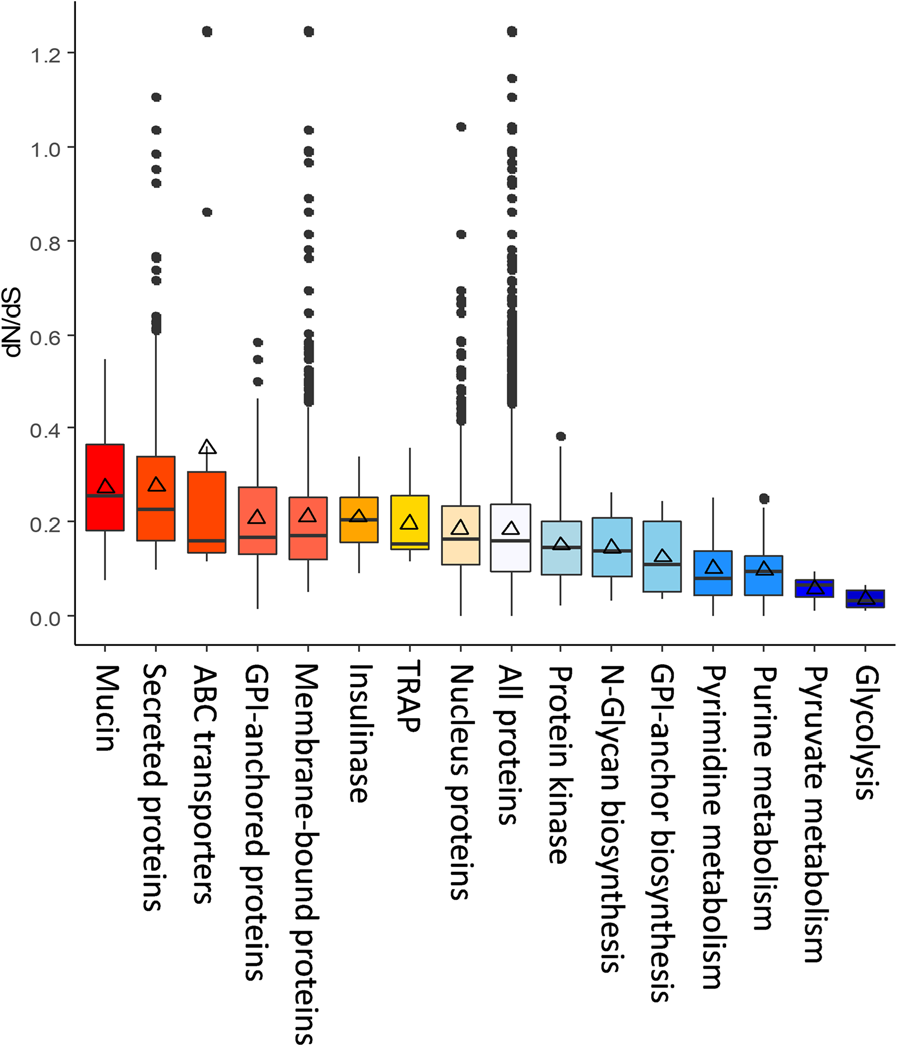
Selective pressure in genes encoding major groups of proteins as indicated by the dN/dS ratios between *Cryptosporidium* chipmunk genotype I and *C. parvum*. Red categories represent groups of proteins with mean dN/dS ratios higher than all proteins in the proteome, while blue categories represent groups of proteins with reduced dN/dS ratios. Triangles: mean dN/dS; horizontal black line: median dN/dS

The dN/dS analysis was used to identify orthologous genes under selection between *Cryptosporidium* chipmunk genotype I and *C. parvum*, two species with different host ranges. Genes encoding invasion-related proteins, secreted proteins, and surface-associated proteins, which could be involved in host immune responses, exhibited elevated dN/dS ratios. In contrast, genes encoding proteins that are involved in metabolic pathways had reduced dN/dS ratios (Fig. 6). Among all orthologous genes, there are only six genes with dN/dS ratios > 1, thus under positive selection. Two of them (*C_ch_8.3686* and *C_ch_8.3664*) encode ABC transporters. Among the 20 orthologous genes with the highest dN/dS ratios, 9 (45%) encode proteins with signal peptides, 11 (55%) encode membrane-bound proteins, and 14 (70%) are located in the subtelomeric regions (Table 4).

**Table 4:** Twenty orthologous genes with the highest dN/dS ratios between *Cryptosporidium* chipmunk genotype I and *Cryptosporidium parvum*

Gene in *Cryptosporidium* chipmunk genotype I	Gene in *C. parvum*	dN/dS ratio	TMHMM	Signal peptide	Subtelomeric location	Annotation
C_ch_8.3686	cgd2_90	1.25	YES	NO	YES	ABC transporter with 9 transmembrane domains and 2 AAA domains
C_ch_8.3664	cgd2_70	1.24	YES	NO	YES	ABC transporter, with 12 transmembrane domains and 2 AAA domains
C_ch_11.460	cgd3_60	1.15	NO	NO	YES	Putative hydrolase
C_ch_105.389	cgd5_4570	1.11	NO	NO	YES	Hypothetical protein with disordered regions
C_ch_10.167	cgd7_640	1.04	NO	NO	YES	Prp16p pre-mRNA splicing factor, HrpA family SFII helicase
C_ch_22.2069	cgd6_3780	1.04	YES	YES	NO	Hypothetical membrane protein with signal peptide and transmembrane domain
C_ch_10.307	cgd8_5370	0.99	YES	NO	YES	Conserved secreted protein
C_ch_37.2969	cgd7_5510	0.99	YES	NO	YES	Secreted protein
C_ch_105.390	cgd6_5490	0.98	NO	YES	YES	Conserved hypothetical protein with low sequence complexity regions
C_ch_1.56	cgd6_50	0.97	YES	NO	YES	Predicted secreted protein
C_ch_50.3279	cgd1_120	0.95	NO	YES	YES	Predicted secreted protein with a cysteine cluster at the C-terminus
C_ch_18.1418	cgd4_2900	0.93	NO	NO	NO	Polyketide synthase
C_ch_19.1673	cgd4_2510	0.92	NO	YES	NO	Predicted secreted protein
C_ch_19.1715	cgd3_2180	0.92	NO	NO	NO	Type I fatty acid synthase
C_ch_35.2958	cgd5_4610	0.89	YES	YES	YES	Conserved secreted protein
C_ch_23.2117	cgd4_1380	0.86	YES	NO	NO	ABC transporter with 2 AAA domains and 14 transmembrane regions
C_ch_50.3280	cgd1_130	0.82	YES	YES	YES	Predicted secreted protein with a cysteine cluster at the C-terminus
C_ch_17.1234	cgd7_3440	0.78	YES	YES	NO	Predicted secreted protein
C_ch_50.3278	cgd1_110	0.77	NO	YES	YES	Predicted secreted protein
C_ch_21.2011	cgd8_40	0.76	YES	YES	YES	Predicted secreted protein of *Cryptosporidium*-specific SKSR gene family
Subtotal	–	–	11/20 (55.0%)	9/20 (45.0%)	14/20 (70.0%)	–

## Discussion

Results of comparative genomic analysis in this study suggest that the metabolic pathways in *Cryptosporidium* chipmunk genotype I are similar to those in major human-infecting *Cryptosporidium* species, including *C. parvum*, *C. hominis*, and *C. meleagridis* [18, 19]. Unlike *C. muris* and *C. andersoni* [12], *Cryptosporidium* chipmunk genotype I does not use the TCA cycle or conventional oxidative phosphorylation for energy production. Like *C. parvum* and *C. hominis*, *Cryptosporidium* chipmunk genotype I possesses an alternative oxidative phosphorylation chain, which is lost in *C. ubiquitum* and *C. baileyi*. The similarity in metabolism between *Cryptosporidium* chipmunk genotype I and other human-infecting species is a reflection of their genetic relatedness. This has been confirmed by results of phylogenetic analyses of 100 conserved proteins and several families of invasion-related proteins.

The genome organization of *Cryptosporidium* chipmunk genotype I is also similar to other intestinal *Cryptosporidium* species. The genome sizes of the human-pathogenic *Cryptosporidium* species are all near 9 Mb, which is slightly smaller than the 9.21 Mb in *C. muris*. As expected, *Cryptosporidium* chipmunk genotype I has a gene content just slightly lower than human-pathogenic *Cryptosporidium* species. In contrast, the genomes of seven *Eimeria* species in chickens vary significantly in size (46.2–69.5 Mb), with the number of predicted protein-encoding genes over a range of ~ 6000–10,000 genes [25]. Similar differences in genome sizes and gene contents exist among *Plasmodium* spp. [26] or *Babesia* spp. [27]. Thus, compared with other apicomplexans, intestinal *Cryptosporidium* species have shown high genome conservation. The differences in host range among intestinal *Cryptosporidium* species could be potentially caused by the minor gene gains and losses or sequence polymorphism in SPDs encoded by genes located in subtelomeric regions.

Compared with *C. parvum*, a major reduction in gene content in *Cryptosporidium* chipmunk genotype I is in the number of subtelomeric genes encoding secreted MEDLE proteins and insulinase-like proteases. *Cryptosporidium parvum* has two subtelomeric genes for insulinase-like proteases (*cgd6_5520–5510* and a paralog of it), compared to one in *Cryptosporidium* chipmunk genotype I (*Cch_105.391*, a paralog of *cgd3_4260*), one (*cgd5/6_5520–5510* ortholog) in *C. meleagridis*, and none in *C. hominis*. The loss of these and some subtelomeric genes encoding secreted MEDLE family proteins in *Cryptosporidium* chipmunk genotype I (6, 2, 2, and 1 copy for *C. parvum*, *C. meleagridis*, *Cryptosporidium* chipmunk genotype I, and *C. hominis*, respectively) may contribute to its narrow host range. In contrast, the number of genes for mucin-type glycoproteins in *Cryptosporidium* chipmunk genotype I is similar to that in human-infecting species. *Cryptosporidium* chipmunk genotype I, *C. hominis*, *C. parvum*, and *C. meleagridis* possess 24 genes encoding mucin-type glycoproteins, whereas gastric species, such as *C. andersoni* and *C. muris*, have lost 16 of them, including those encoding gp60, Muc4, and Muc5, which are important in the attachment and invasion of *C. parvum* [28].

The significance of other gene gains and losses in the genome of *Cryptosporidium* chipmunk genotype I is not yet clear. The gene *Cch_35.2955*, which has three other paralogs in *Cryptosporidium* chipmunk genotype I, was annotated as a new gene at the 3′ end of chromosome 5. *C. parvum* has three orthologs (*cgd5/6_5500*, *cgd6_5500* and *cgd8_10*) while *C. hominis* has six (*Chro.00007*, *Chro.60010*, *Chro.60630*, *Chro.60631*, *Chro.60634* and *Chro.80010*). There is also a loss of the *cgd8_660_670* ortholog in chromosome 8 of *Cryptosporidium* chipmunk genotype I. This gene encodes a large low complexity protein in *C. parvum* and has a paralog (*cgd8_680_690*) downstream. Likewise, *C. hominis* has only one member of this multigene family [11]. In addition, *Cryptosporidium* chipmunk genotype I has lost several other genes, such as orthologs of *cgd4_3690* (encoding a large glycine-rich repeat low complexity protein), *cgd4_4500* (encoding a cysteine-rich protein), *cgd5_2960* (encoding a DEAD/DEAH box helicase), *cgd5_2980* (encoding another DEAD/DEAH box helicase), and *cgd8_4180* (encoding a glycine-rich low complexity protein) in *C. parvum*. Although the functions of these proteins are mostly unknown, these gene losses could contribute to the narrow host range of *Cryptosporidium* chipmunk genotype I.

Most of the highly divergent genes between *Cryptosporidium* chipmunk genotype I and other *Cryptosporidium* spp. encode secreted proteins and half of the highly divergent genes are located in the subtelomeric regions. These secreted proteins could potentially be SPDs in *Cryptosporidium* spp., thus play a role in host specificity of *Cryptosporidium* spp., especially SKSR, FLGN and mucin proteins. Among them, the number of genes encoding SKSR proteins is different between *C. parvum* IIa and IId subtype families, which have different host preference [13]. As in *C. parvum* IId subtype family, 7 paralogous genes encoding SKSR proteins were detected in *Cryptosporidium* chipmunk genotype I, but the sequence of these genes were divergent from those in *C. parvum*. The high sequence diversity of mucin-type glycoproteins between human- and animal-infecting species may also contribute to the host specificity and tissue tropism among *Cryptosporidium* spp. Previously, secretory proteins from dense granules (GRAs), micronemes (MICs), rhoptries (ROPs), and the SRS super-family were identified as potential SPDs in *T. gondii*, which could be responsible for differences in transmission modes, pathogenicity, and host range among *T. gondii* strains [29].

The elevated dN/dS ratios for secreted and surface-associated proteins support their function as SPDs. These proteins are apparently under selection, perhaps as a result of high immune pressure due to their importance in invasion and host-parasite interactions. A similar observation was made in comparative analysis of *C. parvum* and *C. hominis* genomes [30, 31]. Most of the genes with higher dN/dS ratios are located in the subtelomeric regions, supporting the previous conclusion that they undergo more rapid evolution. Three genes encoding ABC transporters are among the top 20 genes with the highest dN/dS ratios between *Cryptosporidium* chipmunk genotype I and *C. parvum*. ABC transporters are “key components of the cellular machinery for endobiotic and xenobiotic detoxification”, thus may contribute to intrinsic drug resistance in *Cryptosporidium* spp. [32]. These genes are expected to be under positive selective pressure. Indeed several ABC transporters were previously identified as highly divergent genes between *C. parvum* IIa (zoonotic) and IIc (anthroponotic) subtype families [33]. Interestingly, two of them, *cgd2_80* and *cgd2_90*, are also within the same region (*cgd2_70* and *cgd2_90*) identified as going through positive selection in the present study. These three ABC transporters encoded by genes within the ABC transporter gene cluster (*cgd2_60* to *cgd2_90*) could be potential targets for drug development.

## Conclusions

*Cryptosporidium* chipmunk genotype I apparently possesses metabolic pathways and invasion-related proteins similar to those in *C. parvum*, *C. hominis*, and *C. meleagridis*. This supports the human-pathogenic nature of *Cryptosporidium* chipmunk genotype I. The loss of two subtelomeric genes of insulinase-like proteases and four genes of secreted MEDLE family proteins compared with *C. parvum* are in agreement with the narrowed host range of *Cryptosporidium* chipmunk genotype I. Sequence differences and selection in genes encoding secreted and surface-associated proteins and ABC transporters could contribute to other biological differences among intestinal *Cryptosporidium* species. More studies on functional genomics and the basic biology of multiple isolates of *Cryptosporidium* chipmunk genotype I are needed to confirm some of the conclusions and improve our understanding of the emerging human pathogen.

## Methods

### Specimen collection and whole-genome sequencing

*Cryptosporidium* chipmunk genotype I isolate 37,763 was collected from one human specimen in Vermont and diagnosed by DNA sequence analysis of the small subunit rRNA gene [34]. Oocysts were purified from the specimen using sucrose and cesium chloride density gradient centrifugations and immunomagnetic separation [35]. The purified oocysts were subjected to five freeze-thaw cycles and overnight digestion with proteinase K. Genomic DNA was extracted from the oocysts by using the QIAamp®DNA Mini Kit (Qiagen Sciences, Maryland, 20,874, USA) and amplified by REPLI-g Midi Kit (Qiagen GmbH, Hilden, Germany). For whole-genome sequencing, 250-bp paired-end reads were generated from the DNA by using Illumina HiSeq 2500 analysis of an Illumina TruSeq (v3) library. After trimming for adapter sequences and poor sequence quality (<phred score less than 25), the sequence reads were assembled de novo by using CLC Genomics Workbench with word size of 63 and bulb size of 500. In a secondary analysis, the genome was also assembled using SPAdes 3.1 (http://cab.spbu.ru/software/spades/).

### Genome structure analysis and gene prediction

An alignment of *Cryptosporidium* chipmunk genotype I genome and published genomes of *C. parvum* IOWA isolate [18], *C. hominis*, *C. ubiquitum* [12], *C. baileyi* [20] and *C. andersoni* [12] was constructed by using Mauve 2.3.1 [36] with default parameters. Circos 0.69 [37] was used to visualize the syntenic relationship (regions with orthologous genes) between the *Cryptosporidium* chipmunk genotype I genome and other four genomes.

AUGUSTUS 3.2.1 [38], Geneid 1.4 [39], and GeneMark-ES [40] were used to predict protein-encoding genes in *Cryptosporidium* chipmunk genotype I with the default settings, after training AUGUSTUS and Geneid with the gene model of the *C. parvum* IOWA genome. Consensus predictor EVidence Modeler [41] was used to generate the gene set based on predictions from the three software packages.

### Functional annotation

The predicted genes of *Cryptosporidium* chipmunk genotype I were annotated by using BLASTP [42] search of the GenBank NR database. Signal peptides and the transmembrane domains were predicted by using SignalP 4.1 [43] and TMHMM 2.0 [44], respectively. GPI-SOM webserver [45] was used to identify proteins with GPI anchor sites. Metabolism analysis was performed using the web server KAAS [46] with the BBH (Bi-directional Best Hit) method and eukaryote gene model. The online databases KEGG (Kyoto Encyclopedia of Genes and Genomes)(http://www.genome.jp/kegg/), Pfam (http://pfam.xfam.org/) [47], and LAMP (Library of Apicomplexan Metabolic Pathways, release-2) [48] were used to annotate catalytic enzymes, functional proteins, and metabolic pathways within the genome.

### Comparative genomics analysis

BLASTP was used for sequence similarity searches among *Cryptosporidium* chipmunk genotype I and other *Cryptosporidium* genomes in CryptoDB (http://cryptodb.org/cryptodb/). Homologous gene families were identified by using OrthoMCL [49]. BLASTP and OrthoMCL were run with e-value thresholds of 1e-3 and 1e-5, respectively. A Venn diagram of shared orthologs and species-specific genes of *C. parvum*, *C. hominis*, *C. ubiquitum*, *C. meleagridis*, and *Cryptosporidium* chipmunk genotype I was drawn using VennPainter (https://github.com/linguoliang/VennPainter). The relationship among proteins in *Cryptosporidium* chipmunk genotype I, *C. parvum*, and *C. meleagridis* was visualized with Gephi (https://gephi.org/) with the Fruchterman-Reingold layout based on the result of BLASTP homology analysis, with threshold of protein pairs sharing 30% identity over 100 amino acids. Comparative analyses of metabolism among *Cryptosporidium* spp. were based on the results of KAAS and data of LAMP. Pfam search results were used in comparisons of transporter proteins and invasion-related proteins among *Cryptosporidium* species. The nonsynonymous to synonymous substitution (dN/dS) ratios between *Cryptosporidium* chipmunk genotype I and *C. parvum* were calculated for orthologous genes using KaKs_Calculator 2.0 [50].

### Phylogenetic analysis

The amino acid sequences of 100 single-copy orthologs shared among *Cryptosporidium* species and *Gregarina niphandrodes* were extracted and concatenated to construct a phylogenetic tree. MUSCLE [51] was used to align the concatenated sequences and with poorly aligned positions being eliminated from the alignment by using Gblocks [52]. Phylogenetic trees based on maximum likelihood (ML) were constructed using RAxML [53] with 1000 replications for bootstrapping. The concatenated sequence from *G. niphandrodes* was used as the outgroup.

## Additional file

Additional file 1: **Table S1.** Gene gains and losses in several *Cryptosporidium* species. **Table S2.** Major putative invasion- and host specificity-associated genes in *Cryptosporidium* spp. **Table S3.** Highly divergent genes among *Cryptosporidium* chipmunk genotype I, *C. parvum* and *C. ubiquitum*. (XLSX 23 kb)

## Abbreviations

AAA: ATPases-associated with diverse cellular activities
ABC: ATP-binding cassette
acetyl-CoA: acetyl-coenzyme A
AOX: Alternative oxidase
ATP: Adenosine triphosphate
dUTP: deoxyuridine triphosphate
FPP: Farnesyl diphosphate
GMP: Guanosine monophosphate
GPI: Glycosylphosphatidylinositol
GRAs: Dense granules
KEGG: Kyoto Encyclopedia of Genes and Genomes
LAMP: Library of Apicomplexan Metabolic Pathways
MICs: Micronemes
OAA: Oxaloacetate
PEP: Phosphoenolpyruvate
PGAP1: Glycosylphosphatidylinositol deacylase
PIG: Phosphatidylinositol glycan
PIG-B: Mannosyltransferase
PNO: Pyruvate: NADP+ oxidoreductase
ROPs: Rhoptries
SPDs: Secreted pathogenesis determinants
TCA: Tricarboxylic acid
TRAPs: Thrombospondin-related adhesive proteins
XMP: Xanthosine 5′-phosphate

## References

[1] Feng Y, Ryan UM, Xiao L (2018). Genetic diversity and population structure of *Cryptosporidium*. Trends Parasitol.

[2] Xiao L (2010). Molecular epidemiology of cryptosporidiosis: an update. Exp Parasitol.

[3] Ryan U, Fayer R, Xiao L (2014). *Cryptosporidium* species in humans and animals: current understanding and research needs. Parasitology.

[4] Silverlas C, Mattsson JG, Insulander M, Lebbad M (2012). Zoonotic transmission of *Cryptosporidium meleagridis* on an organic Swedish farm. Int J Parasitol.

[5] Wang Y, Yang W, Cama V, Wang L, Cabrera L, Ortega Y, Bern C, Feng Y, Gilman R, Xiao L (2014). Population genetics of *Cryptosporidium meleagridis* in humans and birds: evidence for cross-species transmission. Int J Parasitol.

[6] Fayer R, Santin M, Macarisin D (2010). *Cryptosporidium ubiquitum* n. sp. in animals and humans. Vet Parasitol.

[7] Li N, Xiao L, Alderisio K, Elwin K, Cebelinski E, Chalmers R, Santin M, Fayer R, Kvac M, Ryan U (2014). Subtyping *Cryptosporidium ubiquitum*,a zoonotic pathogen emerging in humans. Emerg Infect Dis.

[8] Insulander M, Silverlas C, Lebbad M, Karlsson L, Mattsson JG, Svenungsson B (2013). Molecular epidemiology and clinical manifestations of human cryptosporidiosis in Sweden. Epidemiol Infect.

[9] Lebbad M, Beser J, Insulander M, Karlsson L, Mattsson JG, Svenungsson B, Axen C (2013). Unusual cryptosporidiosis cases in Swedish patients: extended molecular characterization of *Cryptosporidium viatorum* and *Cryptosporidium* chipmunk genotype I. Parasitology.

[10] Guo Y, Cebelinski E, Matusevich C, Alderisio KA, Lebbad M, McEvoy J, Roellig DM, Yang C, Feng Y, Xiao L (2015). Subtyping novel zoonotic pathogen *Cryptosporidium* chipmunk genotype I. J Clin Microbiol.

[11] Guo Y, Tang K, Rowe LA, Li N, Roellig DM, Knipe K, Frace M, Yang C, Feng Y, Xiao L (2015). Comparative genomic analysis reveals occurrence of genetic recombination in virulent *Cryptosporidium hominis* subtypes and telomeric gene duplications in *Cryptosporidium parvum*. BMC Genomics.

[12] Liu S, Roellig DM, Guo Y, Li N, Frace MA, Tang K, Zhang L, Feng Y, Xiao L (2016). Evolution of mitosome metabolism and invasion-related proteins in *Cryptosporidium*. BMC Genomics.

[13] Feng Y, Li N, Roellig DM, Kelley A, Liu G, Amer S, Tang K, Zhang L, Xiao L (2017). Comparative genomic analysis of the IId subtype family of *Cryptosporidium parvum*. Int J Parasitol.

[14] Hunter CA, Sibley LD (2012). Modulation of innate immunity by *Toxoplasma gondii* virulence effectors. Nat Rev Microbiol.

[15] Bouzid M, Hunter PR, Chalmers RM, Tyler KM (2013). *Cryptosporidium* pathogenicity and virulence. Clin Microbiol Rev.

[16] Swapna LS, Parkinson J (2017). Genomics of apicomplexan parasites. Crit Rev Biochem Mol Biol.

[17] Khan A, Shaik JS, Grigg ME (2018). Genomics and molecular epidemiology of *Cryptosporidium* species. Acta Trop.

[18] Abrahamsen MS, Templeton TJ, Enomoto S, Abrahante JE, Zhu G, Lancto CA, Deng M, Liu C, Widmer G, Tzipori S (2004). Complete genome sequence of the apicomplexan, *Cryptosporidium parvum*. Science.

[19] Xu P, Widmer G, Wang YP, Ozaki LS, Alves JM, Serrano MG, Puiu D, Manque P, Akiyoshi D, Mackey AJ (2004). The genome of *Cryptosporidium hominis*. Nature.

[20] Ifeonu OO, Chibucos MC, Orvis J, Su Q, Elwin K, Guo F, Zhang H, Xiao L, Sun M, Chalmers RM, et al. Annotated draft genome sequences of three species of *Cryptosporidium*: *Cryptosporidium meleagridis* isolate UKMEL1, *C. baileyi* isolate TAMU-09Q1 and *C. hominis* isolates TU502_2012 and UKH1. Pathog Dis. 2016;74(7):415–9. 10.1093/femspd/ftw080 PMC5407061 27519257

[21] Putignani L, Possenti A, Cherchi S, Pozio E, Crisanti A, Spano F (2008). The thrombospondin-related protein CpMIC1 (CpTSP8) belongs to the repertoire of micronemal proteins of *Cryptosporidium parvum*. Mol Biochem Parasitol.

[22] Sanderson SJ, Xia D, Prieto H, Yates J, Heiges M, Kissinger JC, Bromley E, Lal K, Sinden RE, Tomley F (2008). Determining the protein repertoire of *Cryptosporidium parvum* sporozoites. Proteomics.

[23] Mauzy MJ, Enomoto S, Lancto CA, Abrahamsen MS, Rutherford MS (2012). The *Cryptosporidium parvum* transcriptome during in vitro development. PLoS One.

[24] Samuelson J, Robbins PW (2015). Effects of N-glycan precursor length diversity on quality control of protein folding and on protein glycosylation. Semin Cell Dev Biol.

[25] Blake DP (2015). *Eimeria* genomics: where are we now and where are we going?. Vet Parasitol.

[26] Rutledge GG, Bohme U, Sanders M, Reid AJ, Cotton JA, Maiga-Ascofare O, Djimde AA, Apinjoh TO, Amenga-Etego L, Manske M (2017). *Plasmodium malariae* and *P. ovale* genomes provide insights into malaria parasite evolution. Nature.

[27] Yamagishi J, Asada M, Hakimi H, Tanaka TQ, Sugimoto C, Kawazu SI (2017). Whole-genome assembly of *Babesia ovata* and comparative genomics between closely related pathogens. BMC Genomics.

[28] O’Connor RM, Burns PB, Ha-Ngoc T, Scarpato K, Khan W, Kang G, Ward H (2009). Polymorphic mucin antigens CpMuc4 and CpMuc5 are integral to *Cryptosporidium parvum* infection in vitro. Eukaryot Cell.

[29] Lorenzi H, Khan A, Behnke MS, Namasivayam S, Swapna LS, Hadjithomas M, Karamycheva S, Pinney D, Brunk BP, Ajioka JW (2016). Local admixture of amplified and diversified secreted pathogenesis determinants shapes mosaic *Toxoplasma gondii* genomes. Nat Commun.

[30] Mazurie AJ, Alves JM, Ozaki LS, Zhou S, Schwartz DC, Buck GA (2013). Comparative genomics of *Cryptosporidium*. Int J Genomics.

[31] Isaza JP, Galvan AL, Polanco V, Huang B, Matveyev AV, Serrano MG, Manque P, Buck GA, Alzate JF (2015). Revisiting the reference genomes of human pathogenic *Cryptosporidium* species: reannotation of *C. parvum* Iowa and a new *C. hominis* reference. Sci Rep.

[32] Zapata F, Perkins ME, Riojas YA, Wu TW, Le Blancq SM (2002). The *Cryptosporidium parvum* ABC protein family. Mol Biochem Parasitol.

[33] Widmer G, Lee Y, Hunt P, Martinelli A, Tolkoff M, Bodi K (2012). Comparative genome analysis of two *Cryptosporidium parvum* isolates with different host range. Infect Genet Evol.

[34] Xiao LH, Escalante L, Yang CF, Sulaiman I, Escalante AA, Montali RJ, Fayer R, Lal AA (1999). Phylogenetic analysis of *Cryptosporidium* parasites based on the small-subunit rRNA gene locus. Appl Environ Microb.

[35] Guo Y, Li N, Lysen C, Frace M, Tang K, Sammons S, Roellig DM, Feng Y, Xiao L (2015). Isolation and enrichment of *Cryptosporidium* DNA and verification of DNA purity for whole-genome sequencing. J Clin Microbiol.

[36] Darling AE, Mau B, Perna NT (2010). Progressive Mauve: multiple genome alignment with gene gain, loss and rearrangement. PLoS One.

[37] Krzywinski M, Schein J, Birol I, Connors J, Gascoyne R, Horsman D, Jones SJ, Marra MA (2009). Circos: an information aesthetic for comparative genomics. Genome Res.

[38] Stanke M, Steinkamp R, Waack S, Morgenstern B (2004). AUGUSTUS: a web server for gene finding in eukaryotes. Nucleic Acids Res.

[39] Parra G, Blanco E, Guigo R (2000). GeneID in drosophila. Genome Res.

[40] Lomsadze A, Ter-Hovhannisyan V, Chernoff YO, Borodovsky M (2005). Gene identification in novel eukaryotic genomes by self-training algorithm. Nucleic Acids Res.

[41] Haas BJ, Salzberg SL, Zhu W, Pertea M, Allen JE, Orvis J, White O, Buell CR, Wortman JR (2008). Automated eukaryotic gene structure annotation using EVidenceModeler and the program to assemble spliced alignments. Genome Biol.

[42] Altschul SF, Gish W, Miller W, Myers EW, Lipman DJ (1990). Basic local alignment search tool. J Mol Biol.

[43] Petersen TN, Brunak S, von Heijne G, Nielsen H (2011). SignalP 4.0: discriminating signal peptides from transmembrane regions. Nat Methods.

[44] Krogh A, Larsson B, von Heijne G, Sonnhammer EL (2001). Predicting transmembrane protein topology with a hidden Markov model: application to complete genomes. J Mol Biol.

[45] Fankhauser N, Maser P (2005). Identification of GPI anchor attachment signals by a Kohonen self-organizing map. Bioinformatics.

[46] Moriya Y, Itoh M, Okuda S, Yoshizawa AC, Kanehisa M (2007). KAAS: an automatic genome annotation and pathway reconstruction server. Nucleic Acids Res.

[47] Finn RD, Bateman A, Clements J, Coggill P, Eberhardt RY, Eddy SR, Heger A, Hetherington K, Holm L, Mistry J (2014). Pfam: the protein families database. Nucleic Acids Res.

[48] Shanmugasundram A, Gonzalez-Galarza FF, Wastling JM, Vasieva O, Jones AR (2013). Library of Apicomplexan metabolic pathways: a manually curated database for metabolic pathways of apicomplexan parasites. Nucleic Acids Res.

[49] Li L, Stoeckert CJ, Roos DS (2003). OrthoMCL: identification of ortholog groups for eukaryotic genomes. Genome Res.

[50] Wang D, Zhang Y, Zhang Z, Zhu J, Yu J (2010). KaKs_Calculator 2.0: a toolkit incorporating gamma-series methods and sliding window strategies. Genomics Proteomics Bioinformatics.

[51] Edgar RC (2004). MUSCLE: multiple sequence alignment with high accuracy and high throughput. Nucleic Acids Res.

[52] Castresana J (2000). Selection of conserved blocks from multiple alignments for their use in phylogenetic analysis. Mol Biol Evol.

[53] Stamatakis A, Ludwig T, Meier H (2005). RAxML-III: a fast program for maximum likelihood-based inference of large phylogenetic trees. Bioinformatics.

